# Enhancement Mechanism of Stibnite Dissolution Mediated by *Acidithiobacillus ferrooxidans* under Extremely Acidic Condition

**DOI:** 10.3390/ijms23073580

**Published:** 2022-03-25

**Authors:** Can Wang, Jin-Lan Xia, Hong-Chang Liu, Yu-Hang Zhou, Zhen-Yuan Nie, Lu Chen, Wen-Sheng Shu

**Affiliations:** 1 Key Laboratory of Biometallurgy of Ministry of Education of China, School of Minerals Processing and Bioengineering, Central South University, Changsha 410083, China; can_wang@csu.edu.cn (C.W.); jlxia@csu.edu.cn (J.-L.X.); yuhangzhou@csu.edu.cn (Y.-H.Z.); zynie@csu.edu.cn (Z.-Y.N.); luchen@csu.edu.cn (L.C.); 2 School of Life Science, South China Normal University, Guangzhou 510631, China; shuwensheng@m.scnu.edu.cn

**Keywords:** stibnite oxidative dissolution, Sb/S speciation transformation, *Acidithiobacillus ferrooxidans*

## Abstract

Oxidative dissolution of stibnite (Sb_2_S_3_), one of the most prevalent geochemical processes for antimony (Sb) release, can be promoted by Sb-oxidizing microbes, which were studied under alkaline and neutral conditions but rarely under acidic conditions. This work is dedicated to unraveling the enhancement mechanism of stibnite dissolution by typical acidophile *Acidithiobacillus ferrooxidans* under extremely acidic conditions. The results of solution behavior showed that the dissolution of Sb_2_S_3_ was significantly enhanced by *A. ferrooxidans*, with lower pH and higher redox potential values and higher [Sb(III)] and [Sb(V)] than the sterile control. The surface morphology results showed that the cells adsorbed onto the mineral surface and formed biofilms. Much more filamentous secondary minerals were formed for the case with *A. ferrooxidans*. Further mineral phase compositions and Sb/S speciation transformation analyses showed that more secondary products Sb_2_O_3_/SbO_2_^−^, Sb_2_O_5_/SbO_3_^−^, SO_4_^2−^, as well as intermediates, such as S^0^, S_2_O_3_^2−^ were formed for the biotic case, indicating that the dissolution of Sb_2_S_3_ and the Sb/S speciation transformation was promoted by *A. ferrooxidans*. These results were further clarified by the comparative transcriptome analysis. This work demonstrated that through the interaction with Sb_2_S_3_, *A. ferrooxidans* promotes S/Sb oxidation, so as to enhance S/Sb transformation and thus the dissolution of Sb_2_S_3_.

## 1. Introduction

Antimony (Sb) is widely used as a metal additive in brake pads, alloys, semiconductors and flame retardants [1,2]. The extensive demand for antimony has promoted the massive mining of antimony ore, leaving behind a large number of abandoned mines and waste tailings, the main sources for the serious Sb pollution around Sb mines areas [1,3,4]. It is known that stibnite (Sb_2_S_3_) is the main form of Sb in the abandoned mines and waste tailings as well as Sb ores, and the oxidative dissolution of Sb_2_S_3_ may be the most predominant cause for serious Sb pollution, the mechanism, however, remains with little known as of yet [5]. It is thus, of great significance to further study and unravel the oxidative dissolution process of stibnite.

Under abiotic conditions, Sb_2_S_3_ can be oxidized slowly by dissolved oxygen possibly by two typical stages, i.e., the dissolution of Sb_2_S_3_ to form aqueous Sb(OH)_3_ and H_2_S (Equation (1)), and the oxidations of Sb(OH)_3_ and H_2_S by dissolved oxygen to form Sb(OH)_5_/Sb(OH)_6_^−^ and sulfate, respectively (Equations (2) and (3)) [6,7]. It was reported that Sb_2_S_3_ oxidation can be significantly promoted under the action of Sb-oxidizing bacteria in nature environments [4,8]. For example, Li et al. isolated six Sb-oxidizing bacteria from Xikuangshan, Hunan, China, the largest Sb ores area in the world, and found that the strain with the highest Sb-oxidation activity could aerobically oxidize 50 mmol/L Sb(III) in 3 days [9]. Nguyen et al. isolated two Sb-oxidizing strains and found they can aerobically oxidize 500 mmol/L Sb(III) within 24 h, and one strain can even anaerobically oxidize Sb(III) with nitrate as the electron acceptor [10]. Xiang et al. demonstrated that the Sb-oxidizing bacteria AS-1 significantly enhanced the oxidations of Sb and S (Equations (4) and (5)), resulting in enhancement of the mobilization of Sb [4]. Up to date, most of the relevant studies were under alkaline, neutral and weak acidic conditions [4,5,11], but rarely under extremely acidic conditions (pH < 3) [1,12].

Sb_2_S_3_ (s) + 6H_2_O (l) ⇋ 2Sb(OH)_3_ (aq) + 3H_2_S (aq) (1)

2Sb(OH)_3_ + O_2_ + 2H_2_O → 2Sb(OH)_5_ (2)

H_2_S + 2O_2_ → H_2_SO_4_ (3)

(4) $${\mathrm{Sb}}_{2}S_{3}\;(s)+6O_{2}\;(\mathrm{aq})+6H_{2}O\;(l)\;\overset{\mathrm{AS}-1}{\to }\;2\mathrm{Sb}(\mathrm{OH}{)}_{3}\;(\mathrm{aq})+3H_{2}{\mathrm{SO}}_{4}\;(\mathrm{aq})$$

(5) $$\mathrm{Sb}(\mathrm{OH}{)}_{3}\;(\mathrm{aq})+3H_{2}O\;(l)\;\overset{\mathrm{AS}-1}{\to }\;\mathrm{Sb}(\mathrm{OH}{{)}_{3}}^{-}\;(\mathrm{aq})+3H^{+}\;(\mathrm{aq})+2e^{-}$$

Of note, is that extremely acidic environments commonly occur for Sb_2_S_3_ when it coexists with other sulfide minerals typically pyrite and arsenopyrite and they are oxidized by acidophiles [1,13]. Torma et al. reported that Sb_2_S_3_ can be oxidized by *Acidithiobacillus ferrooxidans*, the most typical acidophile with a variety of sulfur oxidation pathways (Equations (6)–(8)) [12]. Nguyen et al., however, found that Sb in the contaminated sediment was difficult to solubilize by *A. ferrooxidans* and the extraction efficiency of Sb was the lowest in comparison to those of Cr, Cu, Mn, Ni, and Zn [14]. The reason for that may be the speciation transformation of Sb occurring during the dissolution process of Sb_2_S_3_, i.e., the soluble Sb was transformed into the insoluble Sb, which is unclear as of yet.

(6) $$2S^{0}+3O_{2}+2H_{2}O\;\overset{A.\;f}{\to }\;2{{{\mathrm{SO}}_{4}}^{2}}^{-}+4H^{+}$$

(7) $$S_{2}{{O_{3}}^{2}}^{-}\;\overset{A.\;f}{\to }\;S^{0}+{{{\mathrm{SO}}_{3}}^{2}}^{-}$$

(8) $${{{\mathrm{SO}}_{3}}^{2}}^{-}+H_{2}O\;\overset{A.\;f}{\to }\;{{{\mathrm{SO}}_{4}}^{2}}^{-}+2H^{+}+2e^{-}$$

Therefore, in this work, we focused on the dissolution process of Sb_2_S_3_ mediated by *A. ferrooxidans* under extremely acidic conditions, with a systematic investigation of the Sb and S speciation transformation by Sb and S X-ray photoelectron spectroscopy (XPS) and X-ray absorption near-edge structure (XANES) spectroscopy, besides the determination of the solution parameters, surface morphology and phase composition of the solid residues. We found that under the action of *A. ferrooxidans*, the content of aqueous Sb(OH)_3_ increased significantly, and much of the Sb-oxides (Sb_2_O_3_/SbO_2_^−^, Sb_2_O_5_/SbO_3_^−^) and secondary sulfur products (S^0^, S_2_O_3_^2−^, SO_3_^2−^, SO_4_^2−^) appeared, indicating that by promoting the Sb/S oxidation, *A. ferrooxidans* enhanced the oxidative dissolution process of Sb_2_S_3_. These findings could be of value for deeply understanding Sb occurrence and fate during Sb_2_S_3_ dissolution and providing insights into prevention and control of Sb pollution in acidic Sb mine areas and tailings.

## 2. Results

### 2.1. Physicochemical Parameters

**Figure 1:**
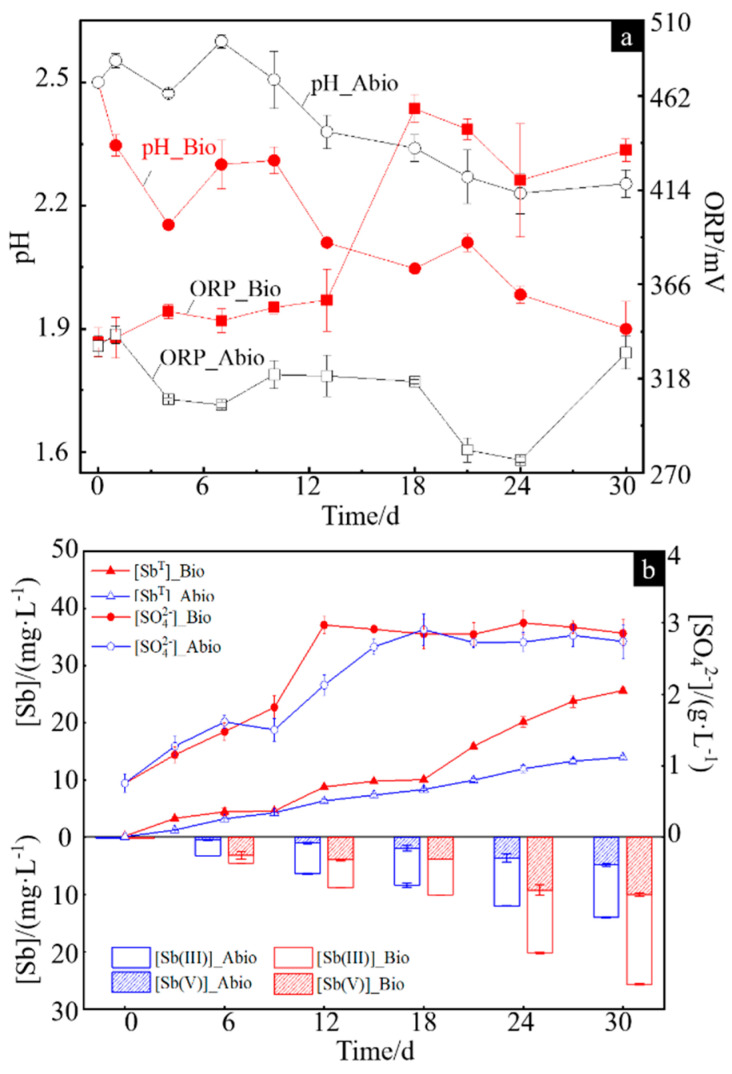
Changes in the pH and ORP values (**a**) and the concentrations of Sb(III), Sb(V), Sb^T^ and SO_4_^2−^ (**b**) in the solution during dissolution of stibnite in the presence or absence of *A. ferrooxidans*.

The physicochemical parameters of the solution for the case with *A. ferrooxidans* and sterile control were in terms of pH, redox potential (ORP), [SO_4_^2−^], [Sb(III)], [Sb(V)] and [Sb^T^] (the concentration of total dissolved Sb), which are shown in Figure 1a,b. The pH values for both cases gradually decreased, and the pH for the case with *A. ferrooxidans* was significantly lower than that in the sterile control (Figure 1a). The ORP values for the sterile control gradually decreased from 335 mV to 274 mV and then increased to 325 mV, while for the case with *A. ferrooxidans,* the ORP values changed slightly on days 0–13, increased sharply by ~100 mV to ~460 mV from day 13 to day 18 and then slightly decreased (Figure 1a). The [SO_4_^2−^] gradually increased to ~4.0 g/L for both cases and then remained basically steady, but it took four days more to reach the steady stage for the sterile control compared to *A. ferrooxidans* (Figure 1b). The [Sb^T^] gradually increased during the whole experiment for both cases, while it was higher for the case with *A. ferrooxidans* than in the sterile control, and the former was approximately two times higher than the latter at the late stage of the experiment (Figure 1b). The concentrations of different forms of dissolved antimony, i.e., [Sb(III)] and [Sb(V)], show a similar trend to [Sb^T^] for both cases. The [Sb(III)] and [Sb(V)] had significant differences on days 0–30 for both cases, while there was only a small [Sb(V)] on days 0–12 for the sterile control (Figure 1b).

### 2.2. Surface Morphology of Solid Residues

**Figure 2:**
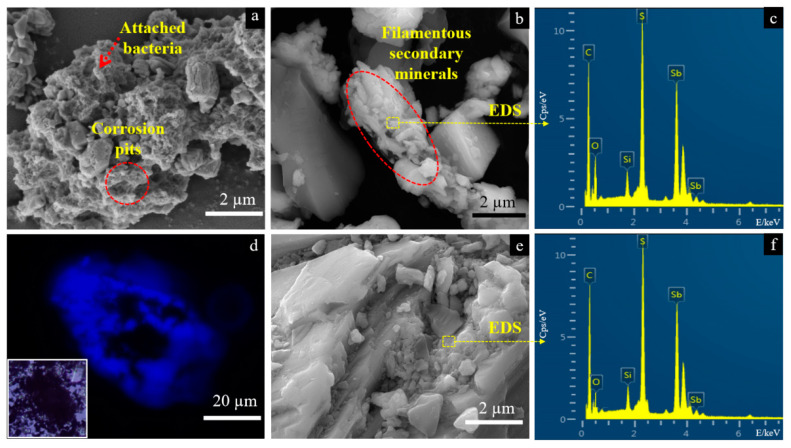
SEM-EDS and FM images of the solid for the group with *A. ferrooxidans* (**a**–**d**) and the sterile group (**e**,**f**) on day 30. Images (**a**,**b**) represent surface morphology of the solid for the group in present of *A. ferrooxidans*, where the dash arrow and circle in image (**a**) shows the attached bacterial cells and corrosion pits, respectively, and dash oval shows the filamentous secondary minerals. Image (**c**) is the elemental composition of the selected area in image (**b**). Image (**d**) represents the biofilm on the solid surface by fluorescence microscopic analysis after 4′,6-diamidino-2-phenylindole (DAPI) staining with DNA, where the square in the lower left corner shows the non-stained image of the same solid particle under the optical microscopy. Images (**e**,**f**) show the surface morphology of the solid for the group in the sterile experiment and the elemental composition of the selected area, respectively.

The surface morphology of solid residues for the case with *A. ferrooxidans* and the sterile control are shown in Figure 2a–f, respectively. During the dissolution of Sb_2_S_3_ in the presence of *A. ferrooxidans*, the bacteria would absorb on the surface of the mineral and form many corrosion pits (Figure 2a). At the same time, there were many filamentous secondary minerals distributed in the culture system, and the EDS results showed that the main elements of the secondary minerals were Sb, S and O (Figure 2b,c). Meanwhile, the attached bacteria on the mineral surface wrapped the solid to generate biofilms, which were bright blue in the fluorescence microscope (FM) image (Figure 2d). Of note, different from the surface of the mineral with the adsorbed bacterial cells (Figure 2a,d), the secondary minerals formed for the case with *A. ferrooxidans* contained no bacterial cells, because no significant N and P elements were detected in the secondary minerals (Figure 2c) and no blue was observed after DAPI staining by FM observation (not shown). In contrast, no significant changes occurred on the surface of Sb_2_S_3_, and the main composition of minerals remained basically steady for the sterile control (Figure 2e,f).

### 2.3. Phase and Composition of Solid Residues

**Figure 3:**
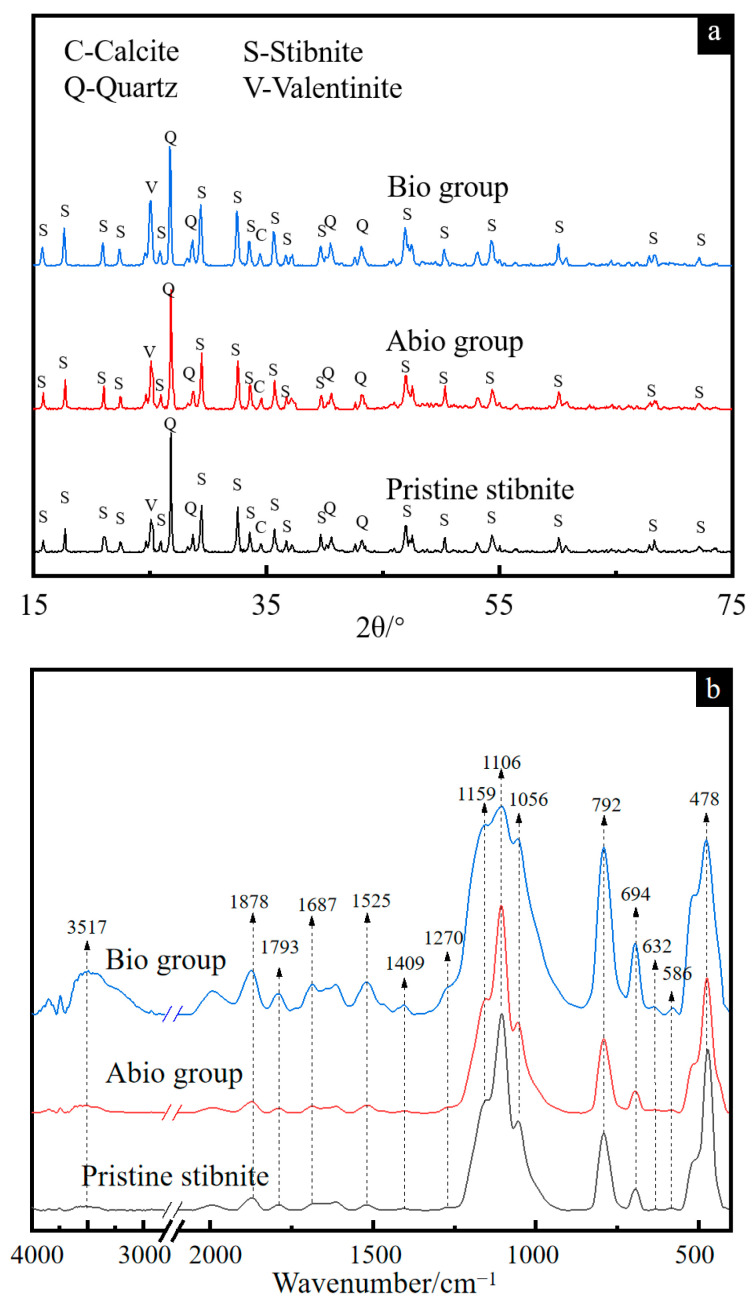
XRD patterns (**a**) and FT-IR spectra (**b**) of pristine stibnite and the solid for the group with *A. ferrooxidans* and the sterile group on day 30.

The XRD patterns of solid residues for the cases with *A. ferrooxidans* and the sterile control are presented in Figure 3a. The results show that the phase types of the solid residues remained unchanged for both cases in comparison with pristine stibnite, comprising Sb_2_S_3_, quartz, Sb_2_O_3_ and calcite, which is consistent with Wu et al. [15]. However, the diffraction signal at 27° associated with Sb_2_O_3_ became stronger for the case with *A. ferrooxidans*, but there was no difference for the case of the sterile control, indicating that more Sb_2_O_3_ was generated [16].

The FT-IR spectra (Figure 3b) show that the bands at 1056, 1106 and 1159 cm^−1^ assigned to the C-O and C-O-C vibrations of polysaccharides became apparently broader for the case with *A. ferrooxidans* than for the sterile control, while the intensities of the bands at 1878, 1793, 1687, 1525 and 1409 cm^−1^ assigned to the C=O and N-H vibrations of proteins were apparently higher for the case with *A. ferrooxidans* [17,18,19,20,21,22], indicating that the bacterial cells were adsorbed on the mineral surface, which is consistent with the FM results (Figure 2d). Notably, strong bands appeared in the range from 3000 cm^−1^ to 4000 cm^−1^ associated with O-H, Sb-S and Sb-O vibrations [23], indicating the enhancement of Sb_2_S_3_ dissolution by *A. ferrooxidans* and the secondary products of antimony oxide(s).

**Figure 4:**
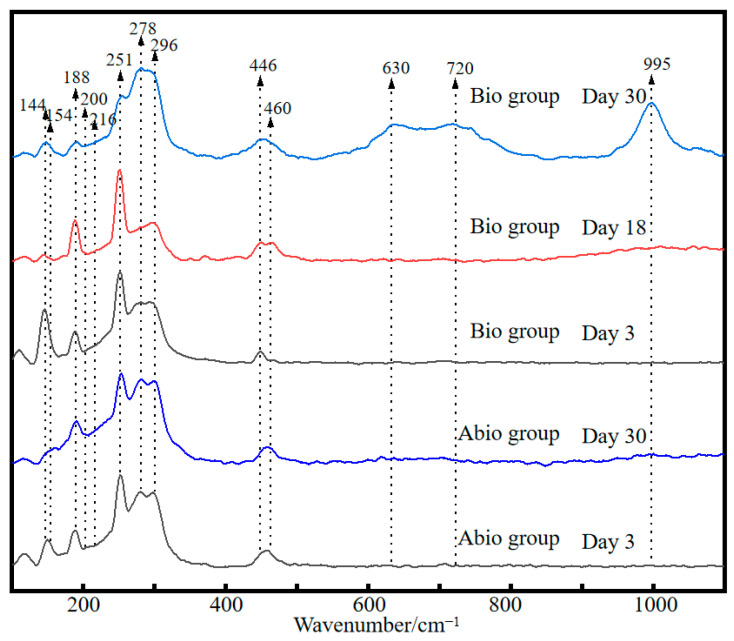
Raman spectra of pristine stibnite and the solid for the group with *A. ferrooxidans* and the sterile group on day 30.

The Raman spectra of the reference samples show significant differences in the intensity and positions of pristine Sb_2_S_3_ mineral and other Sb and/or S-containing compounds (Figure S1), where the bands at 154, 200, 630, 720, and 995 cm^−1^ could be signed as the characteristic peaks of S^0^, Sb_2_O_5_, SbO_3_^−^, Sb_2_O_3_, and SO_4_^2−^, respectively [24,25,26,27]. By comparing the Raman spectra of solid residues for the case with *A. ferrooxidans* and the sterile control (Figure 4) with the reference spectra, strong and broad bands at 154 and 200 associated with S^0^ and Sb_2_O_5_ appeared, respectively, indicating the oxidation of Sb_2_S_3_ and the formation of S^0^ and Sb_2_O_5_. The new bands at 216 and 720 cm^−1^ assigned to Sb_2_O_3_ [28,29] were strongest for the case with *A. ferrooxidans* at day 30, indicating the formation of Sb_2_O_3_ due to the dissolution of Sb_2_S_3_ by *A. ferrooxidans*. Notably, a new band at 630 cm^−1^ assigned to sodium antimonate (NaSbO_3_) [30] appeared for the case with *A. ferrooxidans* rather than the sterile control, indicating of the bio-oxidation of Sb(III) by *A. ferrooxidans*, the formation of Sb_2_O_5_-Sb(V) species and the promotion of Sb_2_S_3_ dissolution by *A. ferrooxidans*. In addition, a new broad band at 995 cm^−1^ assigned to SO_4_^2−^ species appeared at day 30 for the case with *A. ferrooxidans* [31], indicating that the S^2−^ was oxidized to SO_4_^2−^.

### 2.4. S and Sb Speciation Transformation

**Figure 5:**
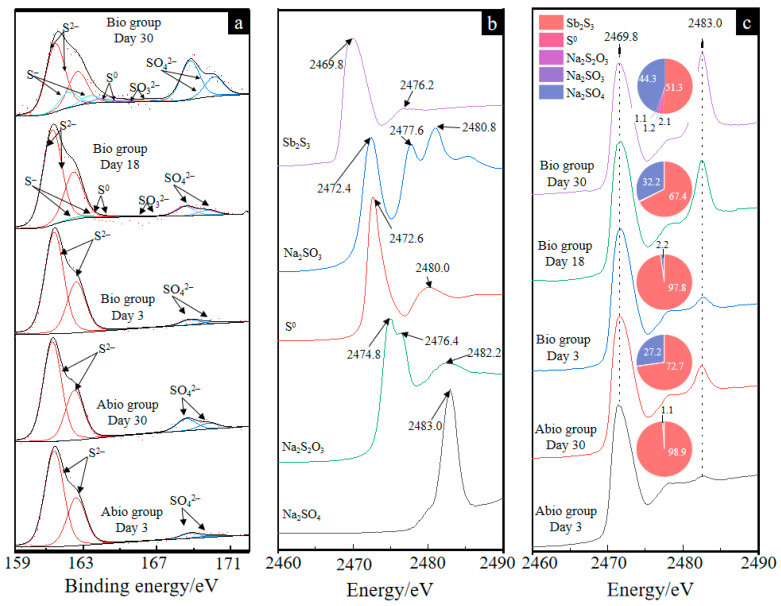
The S 2*p*_(3/2)_ XPS spectra of solid residues (**a**) and the XANES spectra of the reference sample (**b**) and solid residues for the group with *A. ferrooxidans* on days 3, 18 and 30 and the sterile group on days 3 and 30 (**c**). The pie charts in figure (**c**) represent the percentage composition of S species of each XANES spectrum.

The S 2*p*_(3/2)_ XPS spectra and the fitted results of the solid residues for the case with *A. ferrooxidans* at days 3, 18 and 30 and the sterile control at days 3 and 30 are shown in Figure 5a and Table S1, respectively. The results in Figure 5a shows that the surface sulfur species comprised S^2−^ (161.35 eV), S^−^ (eV), S^0^ (164.20 eV), SO_3_^2−^ (166.50 eV), and SO_4_^2−^ (168.50 eV) [32] for the case with *A. ferrooxidans*. The fitted results (Table S1) further show that during bio-oxidation, the content of S^2−^ species gradually decreased from 94.8% to 50.0%, and the content of SO_4_^2−^ species gradually increased from 5.2% to 34.3%. Meanwhile, 11.4% of S^−^, 2.9% of S^0^ and 1.4% of SO_3_^2−^ were detected, indicating that surface S^2−^ of Sb_2_S_3_ was oxidized to SO_4_^2−^ via S^−^, S^0^ and SO_3_^2−^ by *A. ferrooxidans*. In contrast, only S^2−^ and SO_4_^2−^ species were detected in the sterile control, and the content of SO_4_^2−^ species was apparently lower than that in the Bio group, indicating that *A. ferrooxidans* could notably promote the oxidization of sulfur in stibnite.

The S K-edge XANES spectra of the reference samples (Figure 5b) show different peak intensities and positions, which can be used to differentiate the S speciation composition of the unknown sample. It can be seen from Figure 5c that for both the case of *A. ferrooxidans* and the sterile control, the intensity of the peak at 2483.0 eV gradually increased, indicating the formation of SO_4_^2−^, which is consistent with the S 2*p*_(3/2)_ XPS results. According to a previous study [26], the linear combination fitting of the unknown spectra with the reference spectra is suitable to analyze the content of S speciation compositions because of high sensitivity, and the fitted results are shown in Figure 5c. The fitted results of the S K-edge XANES spectra show that Sb_2_S_3_, NaSO_3_, Na_2_S_2_O_3_, S^0^ and Na_2_SO_4_ are detected for both the cases with *A. ferrooxidans* and in the sterile control, and the content of S^2−^ and SO_4_^2−^ species had similar trends to the XPS results, further demonstrating that in the presence of *A. ferrooxidans*, the surface sulfur was oxidized and transformed faster during the bio-oxidation of Sb_2_S_3_. Notably, stibnite, as an acid insoluble mineral, is mainly dissolved by thiosulfate, and the formation of thiosulfate is also found.

**Figure 6:**
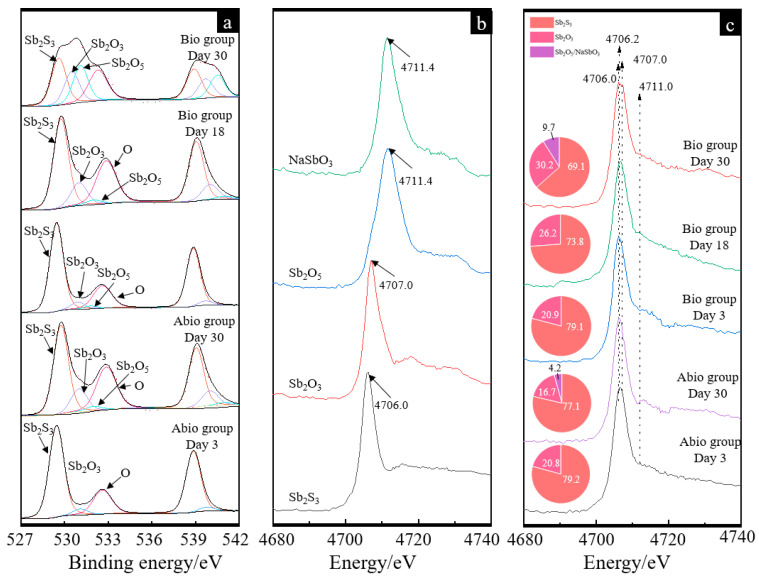
Antimony XPS patterns (**a**), XANES standard and experimental spectra (**b**,**c**) of pristine stibnite and the solid for the group with *A. ferrooxidans* and the sterile group on day 30. The curves in image (**a**) and image (**c**) represent the antimony XPS patterns for the sterile control case on days 3 and 30 and for the case with *A. ferrooxidans* on days 3, 18, and 30, respectively. The pie charts in figure (**c**) represent the percentage composition of Sb species of each XANES spectrum.

According to the Sb 3*d*_(3/2)_ and 3*d*_(5/2)_ XPS spectra (Figure 6a), the surface Sb species comprised Sb_2_S_3_ (529.5 eV; 539.2 eV), Sb_2_O_3_ (532.0 eV; 539.6 eV), and Sb_2_O_5_ (532.1 eV; 540.2 eV) and ranged from an approximate peak O 1*s* (532.6 eV) [33]. The fitted results (Table S2) further show that for the case with *A. ferrooxidans,* the content of Sb_2_S_3_ species gradually decreased from 75.1% to 35.9%, and the content of Sb_2_O_3_ and Sb_2_O_5_ species gradually increased to 22.1% and 25.4%, respectively. In contrast, for the sterile control, 14.2% Sb_2_O_3_ and 3.4% Sb_2_O_5_ were detected.

The Sb L1-edge XANES spectra of the different referenced samples show different peak positions (Figure 6b), where the max adsorption bands for Sb_2_S_3_, Sb_2_O_3_ and Sb_2_O_5_/NaSbO_3_ were 4.706, 4.707 and 4.711 keV, respectively. For the XANES spectra of the case with *A. ferrooxidans* and the sterile control, apparent adsorption bands at 4.706, 4.707 and 4.711 keV were detected, indicating the formation of Sb_2_O_3_ and Sb_2_O_5_/NaSbO_3_. The fitted results (Figure 6c) further show that the Sb species for the case with *A. ferrooxidans* were mainly composed of 76.8–73.8% Sb_2_S_3_ and 20.9–26.2% Sb_2_O_3_ on days 3 and 18, while it became 59.1 Sb_2_S_3_, 30.2% Sb_2_O_3_ and 9.7% Sb_2_O_5_, indicating the oxidation of Sb_2_O_3_ to Sb_2_O_5_. For the sterile control, the Sb species Sb_2_O_3_ and Sb_2_O_5_ were also detected on day 30.

### 2.5. Comparative Transcriptome Analysis

**Figure 7:**
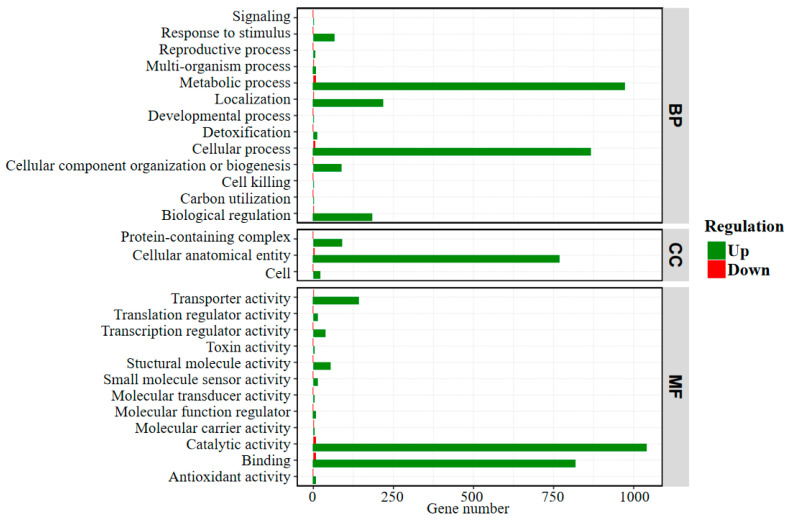
Statistical diagram of the number of differentially expressed genes in GO (gene ontology) Level 2 between the bacterial cells grown on stibnite and S^0^. The GO annotation includes three major functional classifications, i.e., BP: biological process; CC: cellular component; MF: molecular function.

The transcriptomes of bacterial cells grown on stibnite and S^0^ were sequenced and analyzed, which is of value to clarify the enhancement mechanism of the formation of S and Sb-containing intermediates during stibnite dissolution by *A. ferrooxidans* at the transcriptome level. Figure 7 shows the statistical diagram of the number of the differential expression genes between the cells grown on stibnite and S^0^, indicating that the expression of most genes for the bacterial cells grown on stibnite were significantly inhibited in comparison with that on S^0^, which is due to the high toxicity of dissolved Sb(III) and Sb(V) to the bacterial cells during the dissolution of stibnite [11,23]. Of note, there are some genes significantly up-regulated for the cells grown on stibnite than S^0^ (as presented in Table S3), i.e., *AFE_1575* (encoding type I restriction enzyme, R subunit), *AFE_1577* (encoding type I restriction enzyme M protein), *AFE_2393* (encoding transposase), *AFE_3223* (encoding NAD(P)H dehydrogenase, quinone), *AFE_1654* (encoding formate dehydrogenase (FdhD) protein), *AFE_1652* (encoding oxidoreductase alpha subunit), *AFE_1651* (encoding 3-hydroxyisobutyrate dehydrogenase family protein), *AFE_1589* (encoding DNA-damage-inducible protein), *AFE_2312* (encoding Major facilitator superfamily (MFS) transporter), *AFE_1636* (encoding hypothetical protein). These up-regulated genes are mainly annotated as the function of metabolic process, cellular process, binding, catalytic activity, transporter activity, molecular carrier activity and cellular anatomical entity in the GO (gene ontology) level 2, indicating these processes are mostly related to stibnite dissolution by *A. ferrooxidans*.

## 3. Discussion

### 3.1. The Dissolution and Sb/S Intermediates Formation

Previous laboratory experiments have shown that some bacteria in neutral and alkaline environments can accelerate Sb oxidation, reduction and methylation [1,34]. In the present study, we experimentally proved that *A. ferrooxidans* can grow on Sb_2_S_3_ and significantly promote the release of Sb and S of Sb_2_S_3_ and the subsequent oxidation processes in an extremely acidic environment.

The dissolution process of Sb_2_S_3_ is a dissolution equilibrium process, and the dissolution of minerals in the sterile control group is very slow. However, after the addition of *A. ferrooxidans*, the concentrations of H^+^, soluble Sb(III) and Sb(V) in the solution increased significantly (Figure 1). During the dissolution of Sb_2_S_3_, massive amounts of Sb- and S-containing intermediates were released, where the oxidation of S^0^, and ultimately reduced sulfur intermediates to sulfuric acid by *A. ferrooxidans* is one main factor affecting the change in pH [21,35] and the increase in cell density (Figure S2), resulting in a decrease in the pH of the solution (Figure 1a). On the other hand, the attached bacterial cells, evidenced by the FM observation (Figure 2d) and the FT-IR spectroscopy (Figure 3b), can fall off into the culture with the maturation and abscission of biofilms [36], which also results in an increase in the bacterial density of the solution. According to Multani et al., high ORP in the environment promoted Sb(III) oxidation to Sb(V), which could be the main reason for the higher [Sb(V)] for the case with *A. ferrooxidans* than the sterile control (Figure 1b) [3].

The morphology of the intermediates formed in the presence and absence of *A. ferrooxidans* is significantly different, where many filamentous secondary minerals are distributed in the culture system in the presence of *A. ferrooxidans*, while little secondary mineral is formed on the mineral surface in the absence of *A. ferrooxidans*. These intermediates are mainly comprised of S^0^, S_2_O_3_^2−^, SO_4_^2−^, Sb_2_O_3_/SbO_2_^−^, Sb_2_O_5_/SbO_3_^−^ (Figure 5 and Figure 6). According to a previous study, the S_2_O_3_^2−^ was formed due to the oxidation of Sb_2_S_3_ by the thiosulfate pathway, and then oxidized to other S species according to Equations (6)–(8) [12]. Both the solid phases Sb(III)-Sb_2_O_3_ and Sb(V)-Sb_2_O_5_ and the dissolved phases Sb(III)-SbO_2_^−^ and Sb(V)-SbO_3_^−^ were detected, which is probably because the occurrence of Sb_2_O_3_/SbO_2_^−^ or Sb_2_O_5_/SbO_3_^−^ is a dynamic balance process, depending on the pH and ORP of the solution [1]. Of note, during bio-oxidation, the decrease of the content of S^2−^ species and the increase of the content of SO_4_^2−^ and Sb_2_O_5_ species were significantly higher than the sterile control experiment (Figure 5 and Figure 6), indicating that *A. ferrooxidans* significantly promoted the dissolution of Sb_2_S_3_ by enhancing Sb/S transformation.

### 3.2. The Differential Expression Genes Related to Sb_2_S_3_ Bio-Oxidation

Though most of the genes are down-regulated for the bacterial cells grown on stibnite, the bacterial sulfur oxidation activity is still existing evidenced by the expression of the relevant genes, contributing to the rapid formation of reduced sulfur species and the significant increase of [SO_4_^2−^] and decrease of pH in the solution.

The genes with significant up-regulation for the bacterial cells grown on stibnite in comparison with that on S^0^ are mostly related to stibnite dissolution by *A. ferrooxidans*. According to previous studies, *AFE_1652* and *AFE_1654* belong to the cluster that involves the oxidation of formate, which is a basic biochemical process of *A. ferrooxidans*, indicates that the oxidation of formate is probably related to the bio-oxidation stibnite [37,38]. The gene *AFE_3223* is responsible for the biosynthesis of ubiquinone and other terpenoid-quinones, which are electron carriers in electron transfer pathways. According to previous studies, there are two electron transfer pathways proposed in *A. ferrooxidans* for the oxidation of ferrous, by which the one is the “downhill electron pathway” through c-cytochrome Cyc1 to aa3 cytochrome oxidase, and the other is “uphill electron pathway” through c-cytochrome CycA1--> bc1 complex-->ubiquinone pool--> NAD(P) [38,39,40]. The latter pathway has been proved to be related to the Arsenic (III) biotransformation via *A. ferrooxidans* [41], the up-regulation of *AFE_3223* indicates the probable contribution of this pathway to the electron transfer in the biotransformation of Sb (III) in the presence of trace iron. In addition, *AFE_2312* is very similar to xylose and galactose proton symporters, which is proposed to be related to the MFS transporter superfamily contributing to the carbohydrate transporter of the outer membrane [38,42], thus the up-regulation of this gene indicated that the extracellular substances probably took a key role in the stibnite dissolution process and contributes to the biofilm formation of *A. ferrooxidans* on the stibnite surface.

### 3.3. Enhancement Mechanism of Stibnite Dissolution Mediated by A. ferrooxidans

**Figure 8:**
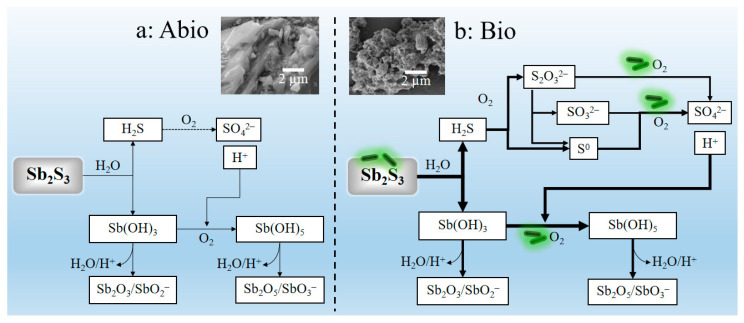
The acidic dissolution mechanisms of Sb_2_S_3_ under abiotic condition (**a**) and biotic condition in the presence of *A. ferrooxidans* (**b**), accompanied with comparison of the dissolution effect (SEM graphs). Where the heavy lines in figure (**b**) were used to demonstrate the enhancement effects of the *A. ferrooxidans* in comparison with the dissolution effects in the abiotic case in figure (**a**).

It can be derived from the above discussion that *A. ferrooxidans* promotes the dissolution of Sb_2_S_3_ most probably by the three following coherent aspects: (i) *A. ferrooxidans* adsorbed on the surface of Sb_2_S_3_ with the up-regulation of genes encoding the MFS transporter, resulting in the formation of biofilms and many corroded pits on the mineral surface with obvious changes in the mineral surface structure and chemical speciation, so as to strengthen the cell-mineral interaction and the dissolution process of minerals; (ii) *A. ferrooxidans* enhanced S oxidation due to the bacterial sulfur oxidation activities, resulting in enhancement of S transformation and cell growth, producing more sulfuric acid to reduce the pH and further enhance the oxidation of Sb(OH)_3_ with Sb transformation forming a large number of Sb_2_O_3_/SbO_2_^−^, Sb_2_O_5_/SbO_3_^−^, and thus accelerating the dissolution of Sb_2_S_3_; (iii) the presence of trace iron enhances the uphill electron transfer to NAD(P), accelerating the transformation of Sb(III) by *A. ferrooxidans*. Moreover, the pertinent enhancement mechanism can be shown schematically in Figure 8. All these findings are of significance to understanding the biogeochemistry of Sb at acidic mining areas and tailings.

## 4. Materials and Methods

### 4.1. Mineral Sample

The original stibnite sample used in this study was from Xikuangshan, Hunan, China. The mineral composition was determined by X-ray fluorescence (XRF, RIGAKU, ZSX Priums, Tokyo, Japan). The X-ray diffraction (XRD) patterns (Figure S3) show that the original Sb_2_S_3_ mainly includes Sb_2_S_3_, Sb_2_O_3_, SiO_2_ and CaCO_3_ phases, and the XRF results (Table S4) show that its composition mainly includes Sb, S and Si. The original Sb_2_S_3_ mineral was dried, ground, passed through −200 mesh screens and retained by −400 mesh screens to obtain particles with sizes of 38 to 75 μm.

### 4.2. Bacterial Strain and Culture Condition

The bacterial strain *A. ferrooxidans* ATCC 23,270 (Accession number of 16S rRNA gene in GenBank: NR_041888) used in this study was provided by the Key Laboratory of Biometallurgy of Ministry of Education of China, Changsha, China. The basal medium for cultivation of *A. ferrooxidans* comprised the following ingredients (in g/L): (NH_4_)_2_SO_4_, 3.0; MgSO_4_, 0.5; K_2_HPO_4_, 0.5; KCl, 0.1; Ca(NO_3_)_2_, 0.01, with an initial pH of 2.5, which is adjusted by 1 M H_2_SO_4_.

### 4.3. Dissolution Experiment

Prior to the biodissolution experiment, the strain was adapted to the energy substrate Sb_2_S_3_ by cultivation for several generations with the addition of 10 g/L Sb_2_S_3_ to the basal medium. The biodissolution experiment of Sb_2_S_3_ by *A. ferrooxidans* was carried out in 250 mL Erlen–Meyer flasks containing 100 mL basal medium and 1 g stibnite (referred to as the Bio group). In contrast, the experiment without *A. ferrooxidans* was taken as the sterile control group (referred to as the Abio group). The initial cell density was 6 × 10^7^ cells/mL. Cultivation of the Bio and Abio groups was performed in an incubator shaker (ZQZY-C8) at 30 °C and 180 r/min.

### 4.4. Analyses Methods

For the culture medium, the pH and redox potential (ORP) values, the cell densities, and the concentrations of SO_4_^2−^ and Sb were determined at 3–5 day intervals during cultivation. The pH of the solution was measured with a pH meter (PHS-3C) by standing the culture for 3–5 min and placing the pH electrode in the solution at 30 °C. Similarly, the ORP was measured with a platinum (Pt) electrode using a calomel electrode (Ag/AgCl) as the reference electrode. The cell density was directly counted by a light microscope (Olympus, Center Valley, PA, USA) with a blood corpuscle counter (XB-K-25). For the [SO_4_^2−^], [Sb(III)] and [Sb(V)], 1 mL of the solution was collected, centrifuged and preserved at −80 °C until analysis. The [SO_4_^2−^] in the solutions was analyzed by using inductively coupled plasma-atomic emission spectroscopy (ICP–OES) (IRIS Intrepid II XSP, Thermo Fisher, Waltham, MA, USA), and the [Sb(III)] was measured by hydride generation (PS Analytical Ltd., Kent, UK) -inductively coupled plasma-atomic emission spectrometry (ICP-AES) (Beijing Haiguang, Beijing, China). Hydride was produced when 2% KBH_4_ (prepared in 0.5% KOH) and 5% HCl were mixed in reducing reagents (5% ascorbic acid + 5% thiourea), and 6 mol/L HCl was added to the solution for the determination of [Sb^T^] [43]. The [Sb(V)] was obtained by subtracting the [Sb(III)] from the [Sb^T^]. The above data were analyzed statistically by Excel 2015 and SPSS 20.0 software and are presented in terms of the mean value with the standard deviation (SD) as the error bar from triplicate cultures (*n* = 3).

For the solid residues, the morphology and composition were analyzed by scanning electron microscopy (SEM) coupled with an energy dispersive spectroscopy (EDS) facility (Oxford AZtecLive Ultim Max 20, Abingdon, UK). Briefly, the samples were first prefixed with 25% formaldehyde overnight, dehydrated using a graded ethanol series, coated with gold and introduced into the SEM chamber for observation. The adsorption of bacterial cells on the mineral surface was observed under a fluorescence microscope (FM) (Nexcope NE900, Ningbo, China) with an intelligent mercury lamp power box (NFP-1N, Ningbo, China) by staining with 4′,6-diamidino-2-phenylindole (DAPI).

The mineral phase and composition were measured by XRD, FT-IR and Raman spectroscopy. Before analysis, the solid samples taken out during cultivation were immediately frozen with liquid N_2_, and then dried under vacuum conditions. The Fourier transform infrared (FT-IR) spectra were collected in the range of 4000–500 cm^−1^ by an FT-IR spectrometer (Nexus 670, Nicolet, Madison, WI, USA). Raman spectra were recorded at room temperature in the range of 200–4000 cm^−1^ by a Raman spectrometer (Thermo Fisher, Sunnyvale, CA, USA). The Sb and S speciation was characterized by Sb and S XPS and XANES spectroscopy. Briefly, XPS spectra were collected by an X-ray photoelectron spectrometer (Thermo Fisher Scientific, East Grinstead, UK) with a voltage and current on X-rays of 12 kV and 6 mA, respectively, and all photoelectron binding energies (BEs) were referenced to the C1 s adventitious contamination peak set at 284.5 eV BE. The Sb L-edge and S K-edge XANES spectra were collected at beamline 4B7A at the Beijing Synchrotron Radiation Facility (BSRF), Beijing, China. The S K-edge XANES spectra were recorded in fluorescence mode from 11,820 eV to 11,980 eV at a step of 0.2 eV and a dwell time of 2 s at each energy level. The Sb L1-edge XANES spectra were recorded in total electron yield (TEY) mode from 4660 eV to 4760 eV at a step of 0.2 eV. Athena software was used for the normalization of XANES spectra and linear combination fitting (LCF) analysis [44].

For the comparative transcriptome analysis, the bacterial cells were grown on the energy substrates S^0^, or stibnite, and were collected at the mid-post logarithmic growth phase. The cell samples for the transcriptome analysis were triplicate (*n* = 3) for each case. The transcriptome was sequenced with an Illumina Hiseq 2500 platform (Illumina, San Diego, CA, USA) by Magigene Co. Ltd., Guangzhou, China. Before sequencing, the total RNA was extracted by Trizol, and then the RNA library was constructed by NEBNextő Ultra II Directional RNA Library Prep Kit for Illumina after removing the ribosomal RNA. The differential expression of genes (DEGs) of *A. ferrooxidans* grown on S^0^ and stibnite were analyzed according to previous studies [45,46,47].

## 5. Conclusions

The dissolution of Sb_2_S_3_ can be enhanced in presence of *A. ferrooxidans* with an obvious decrease in pH and increase in cell density, soluble Sb/S concentration and ORP values, formation of biofilm on the mineral surface with many corroded pits and producing filamentous secondary minerals distributing in the culture system. Comprehensive FT-IR, XRD, XPS, XANES and Raman spectroscopic analyses showed that the main S and Sb speciation in the culture system is S^0^, S_2_O_3_^2−^, SO_4_^2−^ and Sb(OH)_3_, Sb(OH)_5_, Sb_2_O_3_/SbO_2_^−^, Sb_2_O_5_/SbO_3_^−^. It can be inferred that *A. ferrooxidans* accelerated the oxidation of S getting more energy to enhance the cell growth and thus, strengthen the cell-mineral interaction and S transformation; produce more sulfuric acid to promote the oxidation of Sb(OH)_3_ and thus, strengthen Sb transformation. The up-regulated genes related to uphill electron transfer to NAD(P) also contribute to accelerating the transformation of Sb (III) by *A. ferrooxidans*. All the above promote the dissolution of Sb_2_S_3_. All these findings are of significance to understanding the biogeochemistry of Sb at acidic mining areas and tailings.

## Data Availability

Not applicable.

## Supplementary Materials

The following supporting information can be downloaded at: https://www.mdpi.com/article/10.3390/ijms23073580/s1.
