# Supplementary material for: Enhancement Mechanism of Stibnite Dissolution Mediated by Acidithiobacillus ferrooxidans under Extremely Acidic Condition
Source: Int J Mol Sci. 2022 Mar 25;23(7):3580. doi: 10.3390/ijms23073580 (PMC8998812; doi:10.3390/ijms23073580)
Supplement: Supplementary file 1 [file ijms-23-03580-s001.zip › ijms-1643856-supplementary.pdf]

Supplementary materials

for

Enhancement mechanism of stibnite dissolution mediated by *Acidithiobacillus ferrooxidans* under extremely acidic condition

Can Wang [1], Jin-lan Xia [1], Hong-chang Liu [1,*], Yu-hang Zhou [1], Zhen-yuan Nie [1], Lu Chen [1], Wen-sheng Shu [2]

Affiliations:

[1] Key Lab of Biometallurgy of Ministry of Education of China, School of Minerals Processing and Bioengineering, Central South University, Changsha 410083, China; can_wang@csu.edu.cn (C.W.) ; jlxia@csu.edu.cn (J.X.); yuhangzhou@csu.edu.cn (Y.Z.); zynie@csu.edu.cn (Z.N.); luchen@csu.edu.cn (L.C.)

[2] School of Life Science, South China Normal University, Guangzhou 510631, China; shuwensheng@m.scnu.edu.cn (W.S.)

* Correspondence: hongch_liu@csu.edu.cn (H.L.)

The part contains three supplementary figures and four supplementary tables.

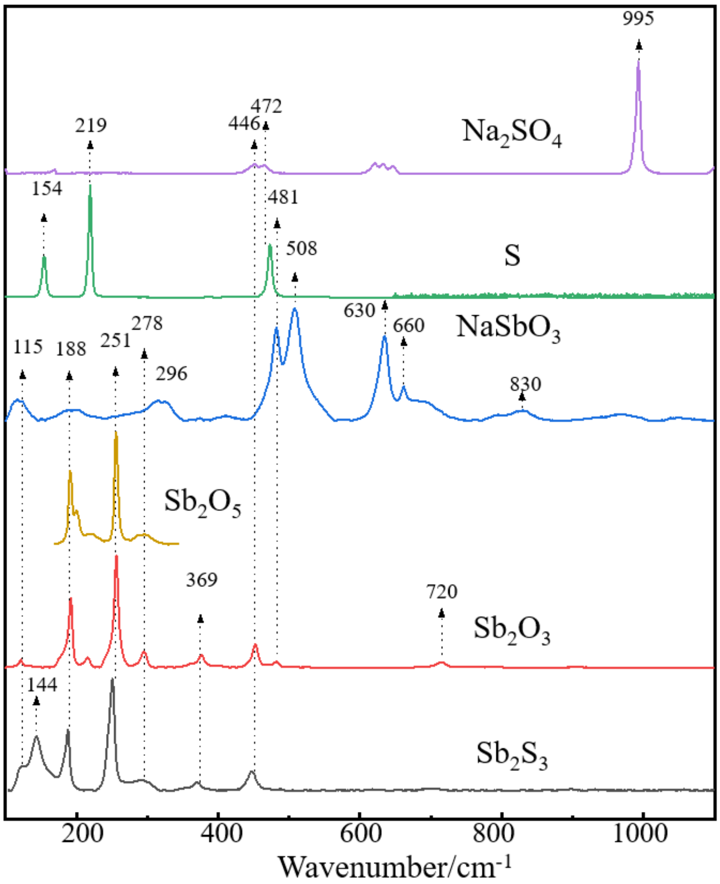

Figure S1. The Raman spectra of reference samples. The Raman spectra of Sb2S3 was completed by the author and Sb2O3, Sb2O5, NaSbO3, S0, Na2SO4 were completed by reference to Roper et al. [1], Yalcin et al. [2], Drewett et al. [3], Bittarello et al. [4], Xia et al. [5], Williams et al. [6], respectively.

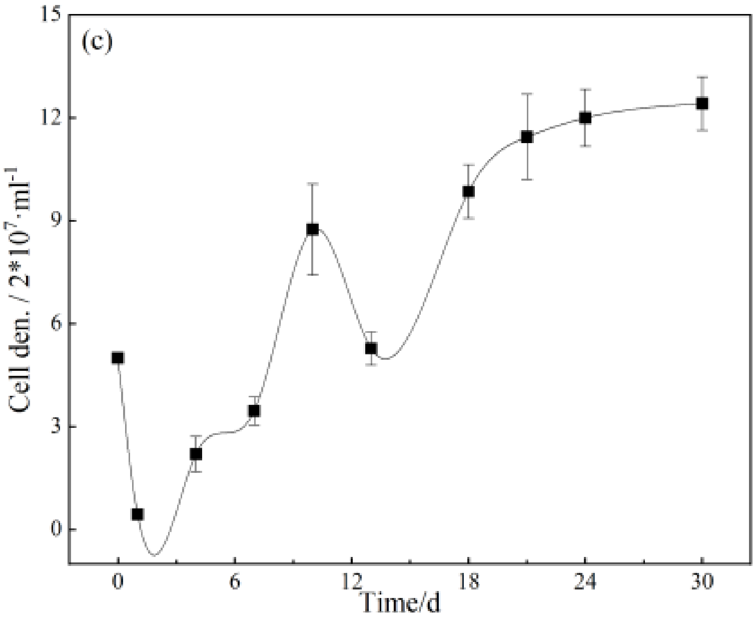

Figure S2. Changes of cell density during dissolution of stibnite.

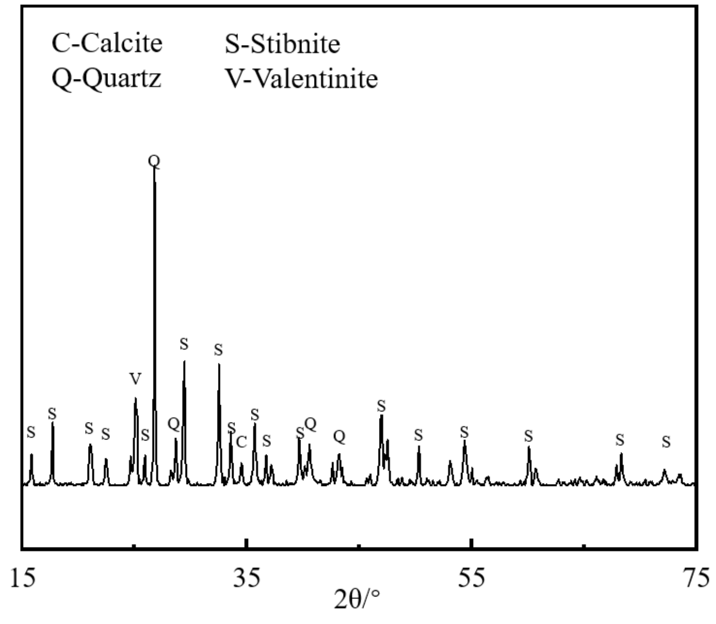

Figure S3. The XRD pattern of pristine stibnite. Where XRD was performed on Cu Kα radiation (40 kV/250 mA) in a RINT2000 vertical goniometer (Bruker D8 Advance, Germany), and the samples were scanned from 5° to 80° 2θ with a step of 0.02° and a dwell time of 4 s.

Table S1. Fitted results of S XPS spectra of stibnite residues for the case with *A. ferrooxidans* (Bio group) at days 3, 18, 30 and in the sterile controls (Abio group) at days 3, 30 with different reference spectra.

Sample		Percentage of contribution of reference samples (%)				
		S2-	S0	S2O32-	SO32-	SO42-
Bio group	3 d	0.0	0.0	0.0	0.0	5.2
	18 d	0.1	0.1	0.1	0.1	9.8
	30 d	2.9	11.4	2.9	1.4	34.3
Abio group	3 d	0.0	0.0	0.0	0.0	5.0
	30 d	0.0	0.0	0.0	0.1	9.2

Table S2. Fitted results of Sb XPS spectra of stibnite residues for the case with *A. ferrooxidans* (Bio group) at day 3, 18, 30 and in the sterile controls (Abio group) at day 3, 30 with different reference spectra.

Sample		Percentage of contribution of reference samples (%)			
		O 1s	Sb2S3	Sb2O3	Sb2O5
Bio group	3 d	15.7	75.1	6.1	3.1
	18 d	21.7	59.6	15.6	3.1
	30 d	16.5	35.9	22.1	25.4
Abio group	3 d	15.5	75.5	6.0	3.0
	30 d	22.2	60.2	14.2	3.4

Table S3. The up-regulated expression of genes in GO level 2 for the bacterial cells grown on stibnite in comparison with that on S^0.

Ontology	GO Annotation	Gene No.	Gene ID
Biological process	Metabolic process	7	AFE_1575, AFE_1577, AFE_2393, AFE_3223, AFE_1654, AFE_1652, AFE_1651
	Cellular process	5	AFE_1589, AFE_1575, AFE_1577, AFE_2393, AFE_1654
	Localization	1	AFE_2312
	Biological regulation	1	AFE_1589
	Multi-organism process	1	AFE_1589
Molecular function	Binding	7	AFE_1589, AFE_1575, AFE_1577, AFE_2393, AFE_3223, AFE_1652, AFE_1651
	Catalytic activity	7	AFE_1575, AFE_1577, AFE_2393, AFE_3223, AFE_1654, AFE_1652, AFE_1651
	Transporter activity	7	AFE_2312
	Molecular carrier activity	7	AFE_1654
Cellular component	Cellular anatomical entity	3	AFE_2312, AFE_1654, AFE_1636

Table S4. The main component (in the form of oxide) of pristine stibnite based on the XRF analysis.

Component	Content (%)
Sb_2O_3	37.56
SO_2	24.50
SiO_2	36.83
Al_2O_3	0.29
WO_3	0.26
Others	0.56

Reference

1. Roper, A.J.; Williams, P.A.; Filella, M. Secondary Antimony Minerals: Phases That Control the Dispersion of Antimony in the Supergene Zone. *Geochemistry.* **2012**, *72*, 9–14, doi:10.1016/j.chemer.2012.01.005.
2. Yalcin, D.; Ozcalik, O.; Altiok, E.; Bayraktar, O. Characterization and Recovery of Tartaric Acid from Wastes of Wine and Grape Juice Industries. *J. Therm. Anal. Calorim.* **2008**, *94*, 767–771, doi:10.1007/s10973-008-9345-z.
3. Drewett, N.E.; Aldous, I.M.; Zou, J.; Hardwick, L.J. In Situ Raman Spectroscopic Analysis of the Lithiation and Sodiation of Antimony Microparticles. *Electrochimica Acta.* **2017**, *247*, 296–305, doi:10.1016/j.electacta.2017.07.030.
4. Bittarello, E.; Cámara, F.; Ciriotti, M.E.; Marengo, A. Ottensite, Brizziite and Mopungite from Pereta Mine (Tuscany, Italy): New Occurrences and Crystal Structure Refinement of Mopungite. *Miner. Petrol.* **2015**, *109*, 431–442, doi:10.1007/s00710-015-0375-5.
5. Xia, J.; Yang, Y.; He, H.; Zhao, X.; Liang, C.; Zheng, L.; Ma, C.; Zhao, Y.; Nie, Z.; Qiu, G. Surface Analysis of Sulfur Speciation on Pyrite Bioleached by Extreme Thermophile *Acidianus Manzaensis* Using Raman and XANES Spectroscopy. *Hydrometallurgy.* **2010**, *100*, 129–135, doi:10.1016/j.hydromet.2009.11.001.
6. Williams, P.A.; Hatert, F.; Pasero, M.; Mills, S.J. IMA Commission on New Minerals, Nomenclature and Classification (CNMNC). *Mineral. mag.* **2014**, *78*, 1241–1248, doi:10.1180/minmag.2014.078.5.10.
